# Cancer Research

**Published:** 1908-04

**Authors:** 


### CANCER RESEARCH.

The Seventh Annual Report of the Work of the Cancer Laboratory of the New York State Department of Health, at Buffalo, shows considerable matter of interest from an experimental standpoint, and it is to be hoped that eventually discoveries of much practical value will emanate from the correllation of the facts observed or confirmed by these enthusiastic scientists.

Especial attention is again called to the evidence bearing on the contagiousness of cancer, which undoubtedly occurs in mice when placed in infected cages, and from the increase in the number of publications bearing upon the so-called "cancer-houses," in which an universal number of individuals contract the disease, they believe this possible method of its spread should not be entirely neglected. Dr. Gaylord therefore urges the Department of Health to recommend to all health officers the desirability of proper sterilization and disposal of all dressings of cancer cases, and the fumigation and sterilization of rooms occupied by cancer patients.

Of the spontaneous cure of cancer, Gaylord and Clowes say that however deep the skepticism may be regarding spontaneous cure of human carcinoma—and this skepticism is certainly widespread,—there is absolutely no doubt of the occurrence of spontaneous cure in mice with cancer, no less than 101 clearly defined cases having come under their observation during the year just passed. The question of spontaneous cure of cancer in human beings is of still greater interest and importance, and although but few authentic cases are to be found in the literature, they acquire an added significance when considered in conjunction with the results on animals.

The conclusions of these authors are that: Spontaneous cure of mice successfully inoculated with Jensen tumor occurs in twenty-three per cent. of the animals.

The chances of such spontaneous cure are inversely proportional to the size of the tumor. The frequency of the occurrence and its distribution in animals suggests that it may be more frequent in human beings than is generally supposed.

The occurrence of spontaneous recoveries from cancer, indicating the existence of immune forces capable of terminating the disease, demonstrates that cancer is not necessarily incurable, and should serve as an additional stimulus to research directed toward the development of a serum therapeutic treatment.

